# Cerebral Small Vessel Disease Outperforms Brain Atrophy as an Imaging Biomarker in Diabetic Retinopathy

**DOI:** 10.1111/1753-0407.70058

**Published:** 2025-02-19

**Authors:** Xinyi Shen, Wen Zhang, Xin Li, Xin Zhang, Qian Li, Min Wu, Linqing Fu, Jiaming Lu, Zhengyang Zhu, Bing Zhang

**Affiliations:** ^1^ Department of Radiology, Nanjing Drum Tower Hospital, Affiliated Hospital of Medical School Nanjing University Nanjing China; ^2^ Institute of Medical Imaging and Artificial Intelligence Nanjing University Nanjing China; ^3^ Medical Imaging Center, Nanjing Drum Tower Hospital, Affiliated Hospital of Medical School Nanjing University Nanjing China; ^4^ Department of Radiology Nanjing Drum Tower Hospital Clinical College of Nanjing University of Chinese Medicine Nanjing China; ^5^ Institute of Brain Science Nanjing University Nanjing China

**Keywords:** brain atrophy, cerebral small vessel disease, diabetic retinopathy, neurodegeneration, type 2 diabetes mellitus

## Abstract

**Aim:**

This study aimed to examine microvascular lesions and neurodegenerative changes in diabetic retinopathy (DR) compared to type 2 diabetes mellitus (T2DM) without DR (NDR) using structural MRI and to explore their associations with DR.

**Methods:**

243 patients with NDR and 122 patients with DR were included. Participants underwent conventional brain MRI scans, clinical measurements, and fundus examinations. Cerebral small vessel disease (CSVD) imaging parameters were obtained using AI‐based software, manually verified, and corrected for accuracy. Volumes of major cortical and subcortical regions representing neurodegeneration were assessed using automated brain segmentation and quantitative techniques. Statistical analysis included *T*‐test, chi‐square test, Mann–Whitney *U* test, multivariate analysis of variance (MANCOVA), multivariate logistic regression, area under the receiver operating characteristic curve (AUC), and Delong test.

**Results:**

DR group exhibited significant differences in 11 CSVD features. Meanwhile, DR showed an atrophy trend in the frontal cortex, occipital cortex, and subcortical gray matter (GM) compared to NDR. After adjustment, DR patients exhibited greater perivascular spaces (PVS) numbers in the parietal lobe (OR = 1.394) and deep brain regions (OR = 1.066), greater dilated perivascular spaces (DPVS) numbers in the left basal ganglia (OR = 2.006), greater small subcortical infarcts (SSI) numbers in the right hemisphere (OR = 3.104), and decreased left frontal PVS (OR = 0.824), total left DPVS (OR = 0.714), and frontal cortex volume (OR = 0.959) compared to NDR. Further, the CSVD model showed a larger AUC (0.823, 95% CI: 0.781–0.866) than the brain atrophy model (AUC = 0.757, 95% CI: 0.706–0.808).

**Conclusion:**

Microvascular and neurodegeneration are significantly associated with DR. CSVD is a better imaging biomarker for DR than brain atrophy.

## Graphical abstract

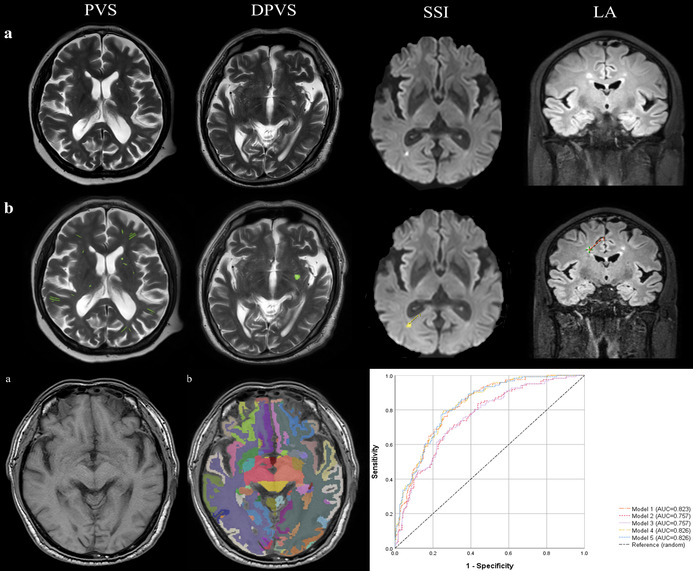

**Summary**

- Significant differences were observed in cerebral small vessel disease (CSVD) and brain atrophy between diabetic retinopathy (DR) patients and non‐diabetic retinopathy (NDR) patients.
- After adjusting for confounding factors, several CSVD parameters showed persistent differences.
- Compared to brain atrophy, CSVD demonstrated a stronger association with diabetic retinopathy.

## 1 Introduction

Type 2 diabetes mellitus (T2DM) is a growing global health issue and is predicted to affect about 700 million people by 2045 worldwide [1]. Diabetic retinopathy (DR) is the most common and most specific microvascular complication of diabetes, with a current global prevalence of 34.6% [2] which is the major cause of preventable blindness in working‐age people [3]. Recent neuroimaging studies have demonstrated that DR adversely affects brain health and increases the risk of dementia [4]. Therefore, indicators are crucial for monitoring brain health in DR patients, especially before the onset of clinical symptoms.

DR is a major microvascular complication of T2DM, and cognitive decline is also a common microvascular complication in this population. Evidence suggests that DR is associated with cognitive decline [5, 6]. The retinal vascular and cerebral small vessels are classic target organs for diabetic microangiopathy [7]. Moreover, the retina is an ontogenetic brain‐derived tissue sharing significant similarities between the retina and cerebral microvasculature in terms of embryological origin, structures, and common physiological characteristics [8, 9]. The retinal microvasculature is therefore regarded as a “window” into the condition of the cerebral microvasculature [10]. Although DR has traditionally been viewed as a disease of the retinal microvasculature, growing evidence over recent years indicates that retinal neurodegeneration is an additional component of diabetic retinal disease [11]. Therefore, there is a strong relationship between retinopathy and brain health. This association may help elucidate the mechanisms linking DR to cognitive impairment, paving the way for improved prediction, prevention, and early intervention of cognitive decline in individuals with DR [12, 13].

CSVD is a common manifestation of cerebral microvascular complications in T2DM. Magnetic resonance imaging (MRI) features of CSVD include recent small subcortical infarcts (RSSI), white matter hyperintensities (WMHs), lacunes (LA), perivascular spaces (PVS), and cerebral microbleeds (CMBs). The total MRI burden of CSVD could be used to comprehensively evaluate the cumulative effect of various types of CSVD. In recent years, numerous studies have demonstrated a significant correlation between CSVD and retinal vascular changes [14, 15]. However, most previous research mainly focused on either the total MRI burden of CSVD or a single marker of the disease, with no studies conducting both quantitative and qualitative analyses of each CSVD marker.

Atrophy in different brain regions conveys distinct information [16]. Therefore, investigating the neurodegeneration in various brain regions is crucial for understanding brain health in individuals with DR. Assessing the volume and volumetric ratios of brain regions allows for the evaluation of the extent of cerebral atrophy. Many previous studies on neurodegeneration have primarily compared individuals with T2DM to non‐diabetic controls [17, 18]. Few previous studies have specifically focused on DR or tried to reveal the differences in neurodegeneration between individuals with DR and those with T2DM without DR (NDR) [19].

The primary objective of this study was to investigate the changes in CSVD and brain volume in DR patients and to explore the associations between DR and these indicators of brain health. We hypothesized that DR is associated with microangiopathy and neurodegeneration, which may provide guidance on the assessment of brain health in DR patients.

## 2 Methods

### 2.1 Participants

The study included 122 individuals with DR matched for age and gender with 243 NDR. The data were entirely derived from clinical electronic records in the Nanjing Drum Tower Hospital inpatient department from January 2012 to June 2022. The inclusion criteria were (1) a confirmed diagnosis of T2DM with laboratory results consistent with the condition (based on the guidelines for diabetes care set by the American Diabetes Association); (2) availability of detailed fundoscopy results during the hospitalizations; and (3) acquisition of brain MRI imaging during the visit. Patients with gestational diabetes, severe acute conditions, admission for diabetic ketoacidosis, a history of diagnosed cataracts or other ocular diseases, or prior ocular treatment were excluded. The research was approved by the Ethics Committee of Drum Tower Hospital Affiliated with Nanjing University Medical School following the Helsinki Declaration and registered at Clinicaltrials.gov (AF/SC‐08/03.0, 2022‐355‐01). Given that this study is a real‐world retrospective analysis, written informed consent could not be obtained from all prior participants; a detailed study protocol was submitted to the Ethics Committee.

### 2.2 Clinical Information and Fundus Examination

Clinical data included demographic information such as age, sex, smoking habit, alcohol consumption, body mass index (BMI), and diabetic duration. Laboratory tests such as total cholesterol (TC), triglycerides (TG), high‐density lipoprotein cholesterol (HDL‐C), low‐density lipoprotein cholesterol (LDL‐C), fasting glucose, and hemoglobin A1c (HbA1c) were collected. Moreover, C‐peptide levels were detected at fasting and 2 h after a standard meal tolerance test. Insulin resistance (IR) was evaluated by fasting C‐peptide (FCP) using the Homeostasis Model Assessment (HOMA) 2 calculator (HOMA2 v2.2.3 http://www.dtu.ox.ac.uk/homacalculator/).

All participants underwent a fundus examination and were subsequently categorized into two groups based on the DR classification standards established by international consensus on clinical DR [20]: DR and NDR groups.

### 2.3 MRI Data Acquisition

MRI scanning was performed on a 1.5‐T or 3.0‐T scanner. The scanning protocol included whole‐brain T_1_‐weighted imaging, T_2_‐weighted imaging, T_2_‐weighted FLAIR imaging, and diffusion‐weighted imaging (DWI). Imaging data were acquired with the parameters that encompassed two‐dimensional (2D) head imaging sequences utilized by all MRI modalities in the hospital over the past decade. Further details are available in the Data S1.

### 2.4 Measurement of CSVD Imaging Features

**FIGURE 1:**
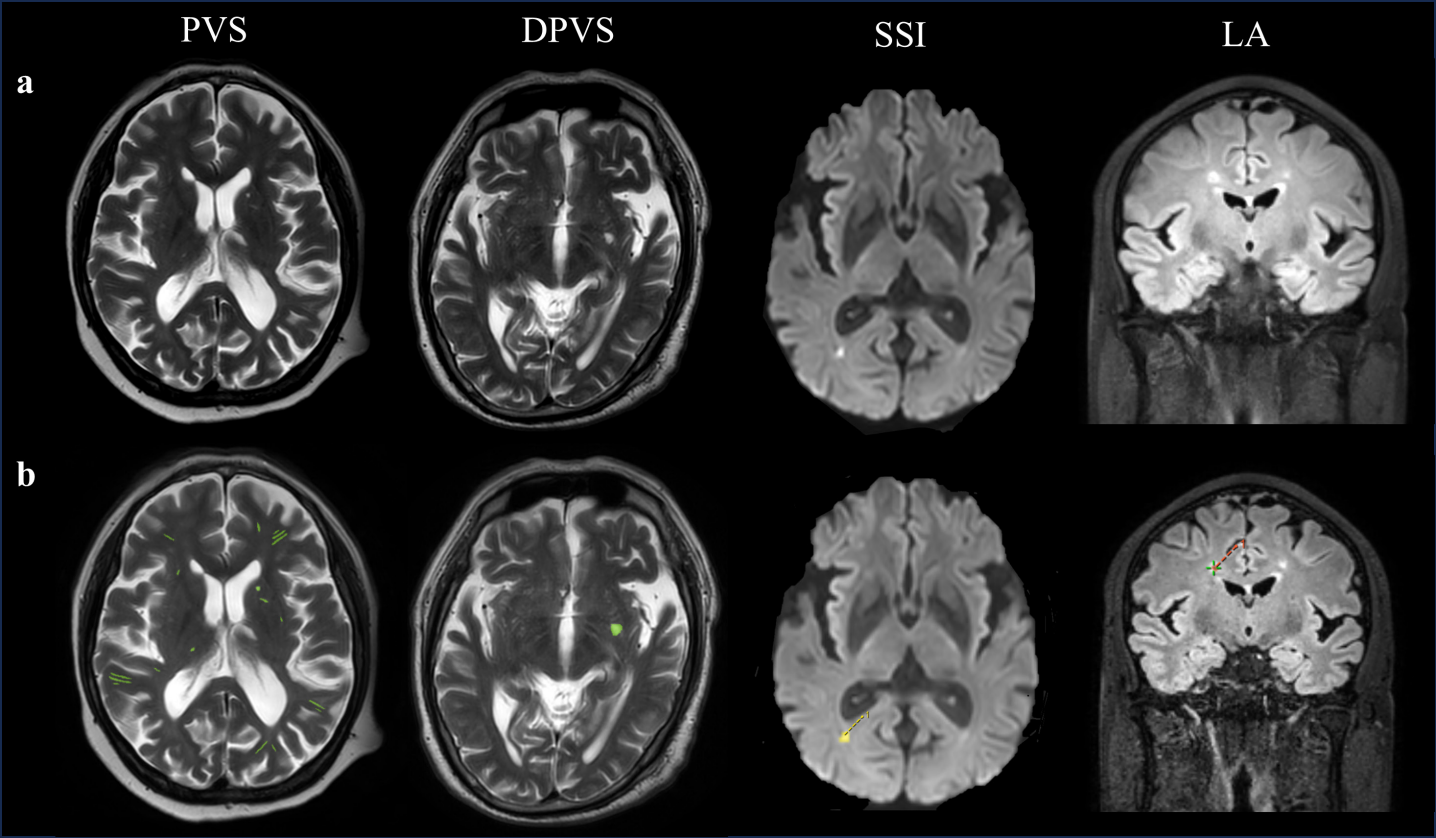
Quantitative steps of CSVD Imaging Features. (a) Original MRI images. (b) The analysis interface of uAI Research Portal.

All MRI scans were processed using an image analysis tool named uAI Research Portal (Shanghai United Imaging Intelligence Co. Ltd.) [21]. Automatic segmentation of RSSI, LA, and PVS was performed using a 2D deep learning model called VB‐Net, constrained by a weighted Dice loss [22, 23]. In the in‐house dataset, the recall for various types of lesions such as RSSI, LA, and PVS reached 87%, 70%, and 74%, respectively. These results were reviewed by two experienced radiologists at our hospital (Figure 1).

T_2_‐FLAIR images were used for WMH segmentation with the software programs MRICRON (University of Nottingham School of Psychology, Nottingham, UK; www.mricro.com) and ITK‐SNAP (University of Pennsylvania, Philadelphia, USA; www.itksnap.org). All scans were checked by visual inspection. First, MRICRON software was used to extract the effective WMH area, and then, ITK‐SNAP software was employed to calculate the WMH volume.

Cerebral microbleeds (CMBs) were not assessable without susceptibility‐weighted imaging (SWI) images. The total MRI burden scores of CSVD ranged from 0 to 3 by combining 3 individual CSVD markers, with 1 point allocated to each marker. The specific criteria for the markers were as follows [24]: (1) WMHs: defined as periventricular or deep brain lesions of varying sizes, hyperintense on T_2_WI or FLAIR imaging, and isointense or hypointense on T_1_WI with abnormal white matter signals. The severity of WMHs was assessed using the Fazekas scale. A score of 3 points for hyperintensities in periventricular white matter or ≥ 2 points for hyperintensities in deep white matter was counted as 1 point; (2) Lacunes: defined as round or oval cerebrospinal fluid‐like signals on T_1_WI and T_2_WI, with a surrounding rim of hyperintensities and central cerebrospinal fluid‐like hypointensities on FLAIR imaging, with a diameter of 3–15 mm, distributed under the cortex. The presence of ≥ 1 lacune was counted as 1 point; (3) PVS: defined as round, oval, or linear lesions that pass through gray or white matter, hypointense on T_1_WI and FLAIR imaging, and hyperintense on T_2_WI. A PVS of level ≥ 2 was counted as 1 point [25]. Perivascular spaces are usually less than 3 mm maximum axial diameter. Dilated perivascular space (DPVS) is a diameter greater than 3 mm. The total CSVD burden score was then categorized into 2 groups based on the simple CSVD score: without burden (0 points) or with burden (1–3 points).

We defined RSSIs according to the STRIVE classification [26] as symptomatic hyperintensity in the territory of one perforating arteriole measuring less than 20 mm in its maximum diameter in the axial plane on DWI. We added RSSI as an individual CSVD marker and named the new variable CSVD + RSSI burden.

### 2.5 Brain Volume Segmentation

**FIGURE 2:**
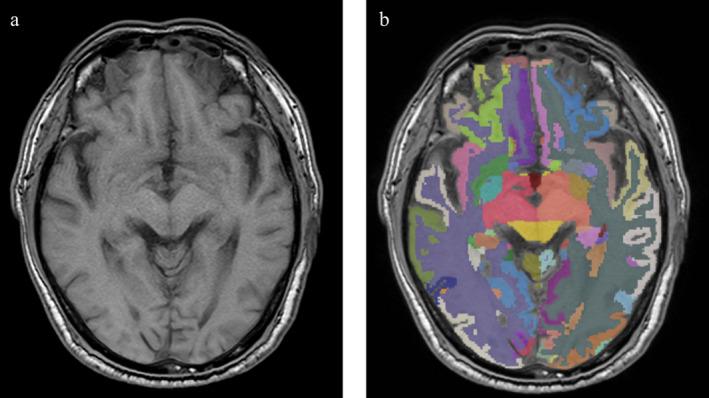
Quantitative steps of the main major cerebral cortex and subcortical nuclei. (a) Original MRI image. (b) The analysis interface of the uAI Research Portal.

2D‐T_1_ images were imported into the uAI Research Portal. Briefly, the preprocessing includes skull stripping, bias correction, and the images were resampled. After that, T_1_ images were segmented for gray matter, white matter, and CSF, and further parcellated into 109 major regions of interest (ROI) according to the Desikan‐Killiany (DK) atlas [27]. The segmentation was done by a pre‐trained cascaded V‐Nets, which combine coarse localization and segmentation refinement and were proven useful in medical image segmentation tasks [28]. Cerebellar regions were not included in our study, and the data were integrated into intracranial volume (ICV) and 12 subregions: frontal cortex, medial temporal cortex, lateral temporal cortex, parietal cortex, occipital cortex, insular cortex, basal ganglia, cerebral white matter, and subcortical gray matter. Volumetric ratios were obtained by dividing the volume by ICV (Figure 2).

### 2.6 Statistical Analysis

Analyses were conducted using SPSS 26.0 and R 3.4.3. Independent sample *t*‐test, Mann–Whitney *U*‐test, and chi‐square test were used to compare differences in clinical Information between the DR and NDR groups. Some CSVD variables (right occipital PVS, thalamus PVS, right lobar DPVS, frontal DPVS, parietal DPVS, temporal DPVS, and all included parameters of SSI and lacunes) were analyzed as categorical variables, which were compared using chi‐square test. In comparison, other continuous variables in CSVD and neurodegeneration were examined using multivariate analysis of variance (MANCOVA). Model 1 adjusted ICV as covariates. Model 2 additionally included age and sex. Model 3 was based on Model 2 with the addition of smoking habits and alcohol consumption. Model 4 further added hypertension and hyperlipidemia. Model 5 incorporated Model 4 with additional adjustments for BMI and duration of diabetes. ICV was not considered a potential confounder when analyzing volumetric ratios, and Model 1 is the unadjusted model, while the other models follow the framework mentioned above. A *p* value < 0.05 after false discovery rate (FDR) correction was considered statistically significant.

Multivariate binary logistic regression was then used to analyze the relationship among DR, CSVD, and brain atrophy. Considering that brain volume is primary data while the volumetric ratio is derived data, we included only brain volume as the indicator of brain atrophy. Model 1 represents the CSVD model. Models 2 and 3 included brain volume parameters, with Model 2 assessing bilateral brain regions and Model 3 focusing on unilateral regions. Models 4 and 5 include microvascular and neurodegeneration parameters, corresponding respectively to models 2 and 3. The association among CSVD variables, brain atrophy variables, and their combinations with DR was determined using receiver operating characteristic (ROC) curve analyses. Differences in area under the curves (AUCs) were assessed using the Delong test.

## 3 Results

### 3.1 Demographics and Clinical Characteristics

A total of 365 T2DM patients (243 NDR and 122 DR) were included in this study. The clinical characteristics of the two groups are shown in Table 1. No significant differences were found in gender, age, vascular risk factors, and serum lipid levels. The DR group had significantly higher HbA1c (*p* = 0.002), longer duration of diabetes (*p* < 0.001), and lower FCR (*p* = 0.006) and 2‐h PCR (*p* < 0.001) than the NDR group. Significant differences were observed in hypertension, diabetic nephropathy, and diabetic peripheral neuropathy between the DR and NDR groups.

**TABLE 1:** Demographic and clinical data of DR group and NDR group.

Index	Total (*n* = 365)	NDR (*n* = 243)	DR (*n* = 122)	*F* or X^2^ or *t*	*p*
**General information**					
Age (years)	60.9 ± 10.0	61.2 ± 10.0	60.4 ± 10.0	0.753	0.452
Sex male (%)	251, 68.8	167, 68.7	84, 68.9	0.001	0.980
**Vascular risk factors**					
Smoking habits (%)	115, 31.5	75, 30.9	40, 32.8	0.139	0.709
Alcohol consumption (%)	57, 15.6	38, 15.64	19, 15.57	3.357	0.067
SBP (mmHg)	136.5 ± 18.8	135.5 ± 18.2	138.5 ± 19.9	−1.420	0.156
DBP (mmHg)	81.2 ± 11.9	81.0 ± 11.1	81.6 ± 13.3	−0.398	0.691
BMI (kg/m^2^)	25.1 ± 2.6	25.2 ± 2.6	25.0 ± 2.7	0.643	0.520
**Diabetes‐related characteristics**					
Duration of diabetes (years)	11.4 ± 7.2	10.0 ± 6.8	14.1 ± 7.2	−5.375	< **0.001**
Nephropathy (%)	22 (6.03)	4 (1.65)	18 (14.75)	23.354	< **0.001**
Peripheral neuropathy (%)	60 (16.44)	20 (8.23)	40 (32.79)	35.656	< **0.001**
FBG (mmol/L)	8.2 ± 2.2	8.0 ± 2.0	8.5 ± 2.4	−1.836	0.067
HbA1C (mmol/L)	8.8 ± 1.9	8.5 ± 1.9	9.2 ± 1.9	−3.182	**0.002**
FCP (mmol/L)	642.9 ± 304.6	673.8 ± 305.0	581.3 ± 295.5	2.761	**0.006**
2‐h PCP (mmol/L)	1643.1 ± 896.7	1788.1 ± 924.2	1354.4 ± 764.5	4.471	< **0.001**
HOMA2 IR	1.62 (1.1, 2.12)	1.64 (1.19, 2.2)	1.46 (0.9, 1.89)	−2.340	**0.019**
**Serum lipid profiles**					
TGs (mmol/L)	1.6 ± 0.9	1.6 ± 0.9	1.5 ± 0.8	1.326	0.186
TC (mmol/L)	4.5 ± 1.1	4.4 ± 1.0	4.5 ± 1.2	−0.363	0.717
HDL cholesterol (mmol/L)	1.1 ± 0.3	1.2 ± 0.3	1.1 ± 0.3	1.060	0.290
LDL cholesterol (mmol/L)	2.6 ± 0.9	2.5 ± 0.9	2.6 ± 1.0	−0.381	0.703
**Complications**					
Hypertension (%)	228 (62.47)	141 (58.02)	87 (71.31)	6.116	**0.013**
Hyperlipidaemia (%)	164 (44.93)	109 (44.86)	55 (45.08)	0.002	0.967
Carotid artery plaques (%)	223 (61.1)	141 (58.02)	82 (67.21)	2.885	0.089
Coronary heart disease (%)	41 (11.23)	29 (11.93)	12 (9.84)	0.359	0.549

*Note:* Data are presented as mean ± SD, median (25th to 75th percentile), or *n* (%) unless otherwise indicated. *p* < 0.05 are highlighted in bold font.

Abbreviations: BMI, Body mass index; DBP, diastolic blood pressure; FBG, fasting blood‐glucose; FCP, fasting C‐Peptide; HbA1c, hemoglobin A1c; HDL‐C, high‐density lipoprotein‐cholesterol; HOMA2‐IR, homeostatic model assessment of insulin resistance; LDL‐C, low‐density lipoprotein‐cholesterol; LDL‐C, low‐density lipoprotein‐cholesterol; PCP, postprandial C‐Peptide; SBP, systolic blood pressure; TC, Serum total cholesterol; TG, triglyceride; WHR, waist‐hip ratio.

### 3.2 CSVD Imaging Features Differences

In the unadjusted model, DR patients had significantly increased burden of left deep DPVS (*p* = 0.001), left basal ganglia DPVS (*p* = 0.002), total right RSSI (*p* < 0.001) right deep RSSI (*p* = 0.002) and right basal ganglia RSSI (*p* = 0.001) than NDR patients after FDR correction (Table 2).

**TABLE 2:** Quantitative analysis of CSVD imaging features of the DR group and the NDR group.

CSVD parameters		Total (*n* = 365)	NDR (*n* = 243)	DR (*n* = 122)	Crude *p*	Model 1	Model 2	Model 3	Model 4	Model 5
						*p*_adj_	*p*_adj_	*p*_adj_	*p*_adj_	*p*_adj_
PVS	Parietal PVS (L)	1 (0, 1)	1 (0, 1)	1 (0, 2)	0.372	0.01*	0.009*	0.006*	0.011*	0.005*
	Temporal PVS (R)	1 (1, 3)	1 (0.5, 2)	1 (1, 3)	0.116	0.028*	0.029*	0.017*	0.033*	0.025*
	Deep PVS	10 (5, 14)	9 (5, 14)	10.5 (7, 16)	0.021*	0.022*	0.022*	0.02*	0.04*	0.05*
	Deep PVS (L)	5 (2, 8)	5 (2, 7)	5 (3, 8.75)	0.054	0.032*	0.033*	0.031*	0.056	0.089
	Deep PVS (R)	5 (2, 8)	4 (2, 7)	5 (3, 8)	0.039*	0.069	0.067	0.054	0.106	0.105
	Basal ganglia PVS (L)	5 (2, 7)	5 (2, 7)	5 (3, 8)	0.047*	0.017*	0.017*	0.017*	0.033*	0.054
	Basal ganglia PVS (R)	5 (2, 7)	4 (2, 7)	5 (3, 8)	0.043*	0.086	0.084	0.066	0.127	0.128
DPVS	Total DPVS (L)	2 (1, 4)	2 (1, 3)	3 (2, 4)	0.010*	0.032*	0.036*	0.033*	0.04*	0.076
	Lobar DPVS (L)	1 (0, 2)	1 (0, 2)	1 (0, 1.75)	0.501	0.026*	0.029*	0.033*	0.053	0.025*
	Deep DPVS	3 (2, 5)	3 (2, 4)	3 (2, 5)	0.023*	0.019*	0.021*	0.02*	0.034*	0.093
	Deep DPVS (L)	2 (1, 3)	1 (1, 2)	2 (1, 3)	0.001*	< 0.001*	< 0.001*	< 0.001*	< 0.001*	0.001*
	Basal ganglia DPVS (L)	2 (1, 3)	1 (1, 2)	2 (1, 3)	0.002*	< 0.001*	< 0.001*	< 0.001*	< 0.001*	0.001*
WMH	Volume (mm^3^)	1158 (0, 10 278)	680 (0, 8663)	2556 (65.5, 11 267)	0.05*	0.31	0.384	0.343	0.355	0.364
	Volumetric ratio	1.89 (0, 16.5)	1.10 (0, 14.0)	3.95 (0.1, 18.9)	0.051	NA	0.028*	0.028*	0.101	0.068
RSSI	Total RSSI	74 (20.27)	40 (16.46)	34 (27.87)	0.011*	NA	NA	NA	NA	NA
	Total RSSI (R)	50 (13.7)	22 (9.05)	28 (22.95)	< 0.001*	NA	NA	NA	NA	NA
	Lobar RSSI (R)	34 (9.32)	17 (7)	17 (13.93)	0.031*	NA	NA	NA	NA	NA
	Deep RSSI (R)	20 (5.48)	7 (2.88)	13 (10.66)	0.002*	NA	NA	NA	NA	NA
	Basal ganglia RSSI (R)	17 (4.66)	5 (2.06)	12 (9.84)	0.001*	NA	NA	NA	NA	NA

*Note:* Some CSVD variables (all included parameters of RSSI) were analyzed as categorical variables while others were treated as continuous variables with a skewed distribution given the data distributions. Model 1 adjusted ICV. Model 2 is model 1 plus adjustment for sex, and age. Model 3 is model 2 plus adjustment for smoking habits and alcohol consumption. Model 4 is model 3 plus adjustment for hypertension and hyperlipidemia. Model 5 is model 4 plus adjustment for BMI and duration of diabetes. The volumetric ratio didn't adjust ICV for it is derived data calculated by dividing the volume by ICV. Only data showing significant differences were presented due to space limitations. Additional details can be found in the Data S1.

Abbreviations: DPVS, dilated perivascular spaces; PVS, perivascular spaces; RSSI, recent small subcortical infarcts; WMH, white matter hyperintensity.

* *p* < 0.05.

MANCOVA was performed to control for confounding variables, and all continuous variables were included in the analysis. Left parietal PVS, right temporal PVS, deep PVS, left lobar DPVS, left deep DPVS, and left basal ganglia DPVS maintained significant differences after adjusting for various parameters in Models 1–5. Additionally, several unstable differences were identified. Significantly more left basal ganglia PVS, left total DPVS, and deep DPVS were observed in DR compared to NDR in Models 1–4. Left deep PVS also showed a significant difference in Models 1–3. After FDR correction, left deep DPVS and left basal ganglia DPVS remained stable significant differences. WMH volume showed a significant difference in the unadjusted Model (*p* = 0.05). WMH volumetric ratio showed significant differences in Models 2 and 3 (both *p* = 0.028). However, these differences were not significant after FDR correction. No significant differences were found in lacunes.

Table 3 shows significant differences in RSSI score (*p* = 0.011) and higher total CSVD + RSSI burden scores (*p* = 0.01) between DR and NDR.

**TABLE 3:** Qualitative analysis of MRI burden of CSVD between the DR group and the NDR group.

Index	Total (*n* = 365)	NDR (*n* = 243)	DR (*n* = 122)	X^2^	*p*
RSSI	74 (20.27)	40 (16.46)	34 (27.87)	6.54	**0.011**
WMH	52 (14.25)	31 (12.76)	21 (17.21)	1.32	0.251
PVS	49 (13.42)	28 (11.52)	21 (17.21)	2.263	0.133
Lacunes	32 (8.76)	18 (7.40)	14 (11.48)	1.680	0.136
Total CSVD burden	93 (25.48)	56 (23.05)	37 (30.33)	3.574	0.059
CSVD + RSSI burden	144 (39.45)	84 (34.57)	60 (49.18)	6.706	**0.010**

*Note:* Data was obtained from chi‐square test. *p* < 0.05 are highlighted in bold font.

Abbreviations: DPVS, dilated perivascular spaces; PVS, perivascular spaces; RSSI, recent small subcortical infarcts; WMH, white matter hyperintensity.

### 3.3 Brain Volume Differences

The results of brain atrophy were presented in Tables 4 and 5. Under the corrected standard, the DR group exhibited significantly smaller gray matter (GM) volumes in the frontal cortex (*p* = 0.01, left: *p* = 0.013, right: *p* = 0.011) and in the right occipital cortex (*p* = 0.024) compared to the controls after adjusting for ICV (Table 4, Model 1). These significant differences remained consistent across Models 2–5. In Models 3–5, the DR group had a reduced occipital cortex volume compared to the NDR group (*p* = 0.04; 0.043; 0.048); the difference showed some volatility. The volumetric ratio of the frontal cortex (both left and right) and right occipital cortex in DR was also significantly larger compared to NDR (Table 5, Model 1–5). Although there was no significant difference in the volumetric ratio of the occipital cortex in the unadjusted Model, a fairly stable significant difference was observed after conducting MANCOVA in Models 2–5 (*p* = 0.046; 0.035; 0.038; 0.047). In Models 2–4, the volumetric ratio of subcortical gray matter (especially left) was significantly decreased in DR compared to NDR. No significant difference remained in the above results after FDR correction.

**TABLE 4:** Brain volume between DR group and NDR group.

Brain volume (cm^3^)	Total (*n* = 365)	NDR (*n* = 243)	DR (*n* = 122)	Model 1	Model 2	Model 3	Model 4	Model 5
				*p*_adj_	*p*_adj_	*p*_adj_	*p*_adj_	*p*_adj_
Frontal	147 ± 14.9	147 ± 14.5	147 ± 15.7	0.010*	0.004*	0.008*	0.013*	0.045*
Frontal (L)	74.2 ± 7.52	74.2 ± 7.29	74.1 ± 7.99	0.013*	0.005*	0.009*	0.016*	0.049*
Frontal (L)	72.7 ± 7.5	72.7 ± 7.38	72.7 ± 7.78	0.011*	0.006*	0.012*	0.018*	0.050*
Occipital	46.1 ± 5.17	46.3 ± 5.34	45.9 ± 4.84	0.051	0.051	0.04*	0.043*	0.048*
Occipital (R)	23.7 ± 2.78	23.8 ± 2.86	23.5 ± 2.61	0.024*	0.023*	0.014*	0.016*	0.026*
Subcortical GM	34.7 ± 4.16	34.7 ± 4.08	34.6 ± 4.33	0.157	0.062	0.071	0.098	0.167
Subcortical GM (L)	17.4 ± 2.22	17.5 ± 2.16	17.4 ± 2.33	0.155	0.055	0.067	0.092	0.126

*Note:* Data was obtained from Independent‐samples *T*‐test and multivariate analysis of variance. Model 1 adjusted ICV. Model 2 is model 1 plus adjustment for sex, and age. Model 3 is model 2 plus adjustment for smoking habits and alcohol consumption. Model 4 is model 3 plus adjustment for hypertension and hyperlipidemia. Model 5 is model 4 plus adjustment for BMI and duration of diabetes. Due to space limitations, data showing significant differences were presented. To ensure consistency between Tables 4 and 5, the analysis data for subcortical GM volume and light subcortical GM volume were also included. Additional details can be found in the Data S1.

* *p* < 0.05.

Abbreviation: GM, gray matter.

**TABLE 5:** Brain volumetric ratios between the DR group and the NDR group.

Brain volumetric ratio (%)	Total (*n* = 365)	NDR (*n* = 243)	DR (*n* = 122)	Model 1	Model 2	Model 3	Model 4	Model 5
				*p*_adj_	*p*_adj_	*p*_adj_	*p*_adj_	*p*_adj_
Frontal	10.5 ± 0.52	10.5 ± 0.52	10.4 ± 0.52	0.014*	0.003*	0.006*	0.01*	0.036*
Frontal (L)	5.3 ± 0.27	5.32 ± 0.27	5.25 ± 0.28	0.017*	0.003*	0.006*	0.012*	0.037*
Frontal (R)	5.19 ± 0.27	5.22 ± 0.27	5.15 ± 0.26	0.02*	0.005*	0.01*	0.015*	0.050*
Occipital	3.3 ± 0.28	3.32 ± 0.28	3.26 ± 0.29	0.061	0.046*	0.035*	0.038*	0.047*
Occipital (R)	1.7 ± 0.15	1.71 ± 0.15	1.67 ± 0.16	0.031*	0.023*	0.013*	0.015*	0.026*
Subcortical GM	2.48 ± 0.25	2.49 ± 0.25	2.45 ± 0.24	0.107	0.026*	0.03*	0.043*	0.086
Subcortical GM (L)	1.25 ± 0.14	1.26 ± 0.13	1.23 ± 0.14	0.113	0.026*	0.031*	0.044*	0.069

*Note:* Data was obtained from Independent‐samples *T*‐test and multivariate analysis of variance. Model 1 is the unadjusted model. Model 2 is model 1 plus adjustment for sex and age. Model 3 is model 2 plus adjustment for smoking habits and alcohol consumption. Model 4 is model 3 plus adjustment for hypertension and hyperlipidemia. Model 5 is model 4 plus adjustment for BMI and duration of diabetes. Only data showing significant differences were presented due to space limitations. Additional details can be found in the Data S1.

Abbreviation: GM, gray matter.

* *p* < 0.05.

### 3.4 Correlations Among DR, CSVD, and Brain Atrophy

Multivariate logistic regression analysis was performed to further evaluate the associations among DR, CSVD, and brain atrophy. In Model 1, Left frontal PVS (OR 0.831, *p* < 0.001), left parietal PVS (OR 1.39, *p* = 0.011), deep PVS (OR 1.063, *p* = 0.014), total left DPVS (OR, *p* = 0.044), left basal ganglia DPVS (OR 2.019, *p* < 0.001) and total right RSSI (OR 2.977, *p* = 0.004) were independently associated with DR. We found that participants with DR were associated with lower GM volume in frontal cortex volume (OR 0.964, *p* = 0.005, especially right, OR 0.935, *p* = 0.003) in Models 2 and 3. Left frontal PVS (OR 0.824, *p* < 0.001), total left DPVS (OR 0.714, *p* = 0.032), and frontal cortex volume (OR 0.959, *p* = 0.036) were negatively correlated with DR, and left parietal PVS (OR 1.394, *p* = 0.012), deep PVS (OR 1.066, *p* = 0.01), left basal ganglia DPVS (OR 2.006, *p* < 0.001), and total right RSSI (OR 3.104, *p* = 0.003) were positively correlated with DR in Model 4. Similar results were obtained in Model 5 (Table 6).

**TABLE 6:** Multivariate analysis of CSVD and brain atrophy.

Variable	Model 1		Model 2		Model 3		Model 4		Model 5	
	OR (95% CI)	*p*	OR (95% CI)	*p*	OR (95% CI)	*p*	OR (95% CI)	*p*	OR (95% CI)	*p*
ICV (m^3^)	NA	NA	1.005 (1.001, 1.009)	0.024	1.005 (1.001, 1.009)	0.012	1.006 (1.001, 1.01)	0.021	1.005 (1.001, 1.01)	0.019
Frontal PVS (L)	0.831 (0.751, 0.92)	< 0.001	NA	NA	NA	NA	0.824 (0.743, 0.913)	< 0.001	0.824 (0.743, 0.914)	< 0.001
Parietal PVS (L)	1.39 (1.077, 1.794)	0.011	NA	NA	NA	NA	1.394 (1.077, 1.804)	0.012	1.395 (1.078, 1.804)	0.011
Deep PVS	1.063 (1.012, 1.115)	0.014	NA	NA	NA	NA	1.066 (1.015, 1.12)	0.01	1.067 (1.016, 1.121)	0.01
Total DPVS (L)	0.735 (0.544, 0.992)	0.044	NA	NA	NA	NA	0.714 (0.526, 0.971)	0.032	0.715 (0.526, 0.972)	0.032
Basal ganglia DPVS (L)	2.019 (1.354, 3.009)	< 0.001	NA	NA	NA	NA	2.006 (1.34, 3.003)	< 0.001	2.028 (1.354, 3.039)	< 0.001
Total RSSI (R)	2.977 (1.419, 6.245)	0.004	NA	NA	NA	NA	3.104 (1.465, 6.579)	0.003	3.057 (1.443, 6.478)	0.004
Frontal (cm^3^)	NA	NA	0.964 (0.93, 1.000)	0.005	NA	NA	0.959 (0.922, 0.997)	0.036	NA	NA
Frontal (R) (cm^3^)	NA	NA	NA	NA	0.935 (0.872, 1.002)	0.050	NA	NA	0.922 (0.857, 0.993)	0.033

*Note:* Data was obtained from multivariate binary logistic regression models after adjusting for confounding factors such as age, hypertension, duration of diabetes, HbA1C, and 2‐h PCP. Model 1 represents the CSVD model. Models 2 and 3 included brain volume parameters, with Model 2 assessing bilateral brain regions and Model 3 focusing on unilateral regions. Models 4 and 5 include both microvascular and neurodegeneration parameters, corresponding respectively to models 2 and 3.

Abbreviations: CI, confident interval; DPVS, dilated perivascular spaces; OR, odds ratio; PVS, perivascular spaces; RSSI, recent small subcortical infarcts.

**FIGURE 3:**
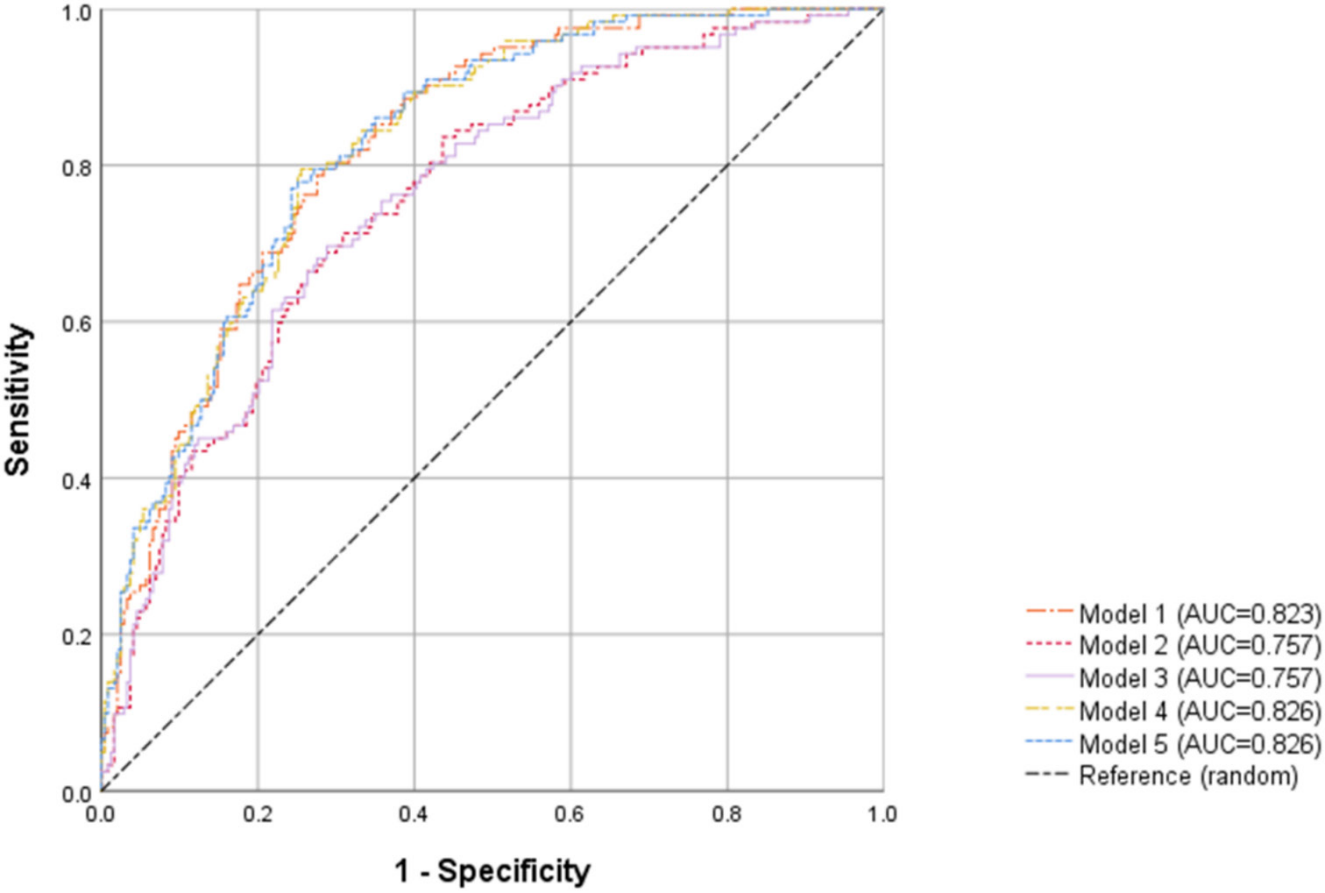
ROC curve showing AUCs for Model 1–5 based on the presence of DR. Model 1 includes the CSVD parameters. Models 2 and 3 include the volumes of bilateral brain regions and unilateral brain regions, respectively. Models 4 and 5 include both CSVD and brain atrophy parameters, and model 4 uses bilateral brain region volumes as markers for brain atrophy while model 5 replaces them with unilateral brain region volumes.

Figure 3 showed the AUC of 5 models. The AUC (95% CI) was 0.823 (0.781, 0.866) for CSVD (Model 1) and 0.757 (0.706, 0.808) for brain atrophy (Model 2, 3). When combining CSVD and brain atrophy (Model 4, 5), the AUC was 0.826 (0.784, 0.868). DeLong's test confirmed that Model 1 had significantly better performance than Models 2 and 3 (both *p* = 0.023).

## 4 Discussion

In this study, we combined quantitative analysis and qualitative analysis to assess MRI markers including CSVD and brain gray matter volume in patients with DR and NDR. Our findings showed that: (1) Significant differences in CSVD, mainly including PVS, DPVS, and RSSI parameters, were observed between the DR and NDR. (2) Approximately significant atrophy in the frontal cortex, occipital cortex, and subcortical GM was found in the DR group compared to the controls. (3) PVS, DPVS, RSSI in specific local regions and frontal cortex volume were independently associated with DR. The CSVD model showed a higher AUC than brain atrophy models, suggesting that CSVD may serve as a more reliable biomarker for DR.

The present study confirmed the changes in CSVD markers in DR patients. Previous research has shown DR was correlated with the total MRI burden of CSVD in T2DM patients [14]. Higher basal ganglia PVS (BG‐DPVS) severity and higher total cerebral SVD score have been found such that with the aggravation of DR, BG‐DPVS severity [29] and total CSVD tended to increase, consistent with our study. Given the large voxel size of our clinical routine scans, we abstained from lesion volumetrics and rather categorized them into PVS and DPVS based on a delineation criterion of 3 mm. PVS has been proposed to be a part of a macroscopic clearance mechanism called the glymphatic system [30], which may be related to the development of neurodegenerative diseases. Previous studies suggest that PVS has the potential to dilate and accelerate waste clearance, and the enlargement of PVS was proposed to represent the blockage of brain drainage pathways [23, 31]. Although the mechanism underlying enlarged PVS is not completely understood, previous studies have shown that associations of disease with visible PVS differ based on their location [32]. The BG‐DPVS burden has been associated with hypertensive angiopathy, systemic markers of inflammation, lacunar stroke, and vascular cognitive impairment [33]. In CSVD, several studies have demonstrated that higher DPVS counts (mostly located in the basal ganglia) are related to cognitive impairment [34]. In our study, left basal ganglia DPVS severity was associated with DR, suggesting that DR is associated with systemic markers of inflammation and vascular cognitive impairment. We also found changes in PVS burden in the left parietal, deep white matter, and left frontal regions, which were not predilection sites of PVS, and previous studies have not evaluated the PVS burden in these areas. We speculate that the abnormalities of PVS in these regions may indicate a dysfunction in the cerebrospinal fluid clearance pathways. Our findings may provide novel perspectives for the explanation of the pathogenesis underlying brain manifestations in DR patients.

Our study found that DR was related to the burden of CSVD+RSSI, and the more severe the DR was, the higher the CSVD+RSSI score was. The burden of CSVD demonstrated similar trends, although the result was not significant (*p* = 0.059). We observed a significant correlation between RSSI and DR, which further enriched our understanding of DR. A previous neuroimaging study has reported that diabetes is a risk factor for brainstem RSSIs, while no impact of diabetes on subcortical white matter and basal ganglia region RSSIs was found [35]. So far, knowledge about baseline clinical or morphological correlates associated with RSSI is scarce, while several reports have investigated lesion evolution of RSSI, which should be assessed in the future by follow‐up neuroimaging.

Increased lacunes and WMH burden in T2DM have been reported by several studies [36]. As a result, we further investigated the changes in lacunes and WMH between DR and NDR in the current study, but no significant difference in lacunes has been found, and there were only unstable differences in the volumetric ratio of WMH. We suggest that lacunes and WMH contribute less than PVS and RSSI to the brain changes in DR.

Atrophy trends in the frontal cortex, right occipital cortex, and subcortical GM have been found in this study. The frontal lobe, which plays a key role in higher‐order cognitive functions, is a crucial brain region strongly linked to overall brain health [37]. The occipital cortex, primarily involved in visual information processing, is also important in DR [38]. A prior study has demonstrated that cortical regions involved in early visual processing may be affected in diabetic patients even before retinal damage occurs [39]. Another MRI study has revealed that worse glycemic control was significantly correlated with subcortical GM atrophy, which is associated with cognitive impairment in adults with diabetes [40]. These findings may suggest a potential link between neurodegeneration and cognitive impairment in diabetes in DR. Nonetheless, no significant differences in brain atrophy remained after FDR correction. The lack of differences may be attributable to a large number of included parameters, which makes it difficult to pass the FDR correction when the effect size is not very strong. However, the use of standard clinical imaging sequences improves data accessibility for research purposes. We have introduced a novel algorithm for automatic brain segmentation and quantitative analysis, which can be widely applied in clinical imaging, offering new opportunities for future clinical research.

Some limitations in this study should be considered. First, this is a cross‐sectional study conducted while T2DM is a chronic disease, and the effect on blood vessels and nerves is a gradual and progressive process. Therefore, large longitudinal studies are needed to clarify the impact of DR on brain health. Second, given the limited participants, the categorization was solely based on the presence or absence of DR, without further stratification by DR grades, making it difficult to extrapolate these results to a wider population. Third, MRI scanning parameters of image data were inconsistent, which may introduce variability in the results.

In conclusion, our study reveals the association among DR, CSVD, and brain atrophy. Additionally, microvascular lesions serve as a more reliable imaging biomarker for DR than neurodegeneration. This study provides imaging evidence for the influence on brain health in DR and helps to further understand the association between cognitive impairment and DR, so as to gain early intervention opportunities for patients to protect brain health.

## Author Contributions

B.Z. was responsible for the conception and design of the study. X.S., X.L., Q.L., and M.W. conducted the study and participated in data collection. W.Z., L.F., and Z.Z. were responsible for statistical considerations in the analysis. X.S. was responsible for writing the first draft. J.L., X.Z., and W.Z. participated in critically reviewing and interpreting the data for the manuscript. All authors had full access to all of the study data and shared the final responsibility for the decision to submit this report for publication.

## Conflicts of Interest

The authors declare no conflicts of interest.

## Supporting information


**Data S1** Supporting Tables.

## Data Availability

The datasets generated and analyzed during the current study are not publicly available because of the need for ethics and regulatory approvals, but are available from the corresponding author upon reasonable request.
