# Supplementary material for: Cerebral Small Vessel Disease Outperforms Brain Atrophy as an Imaging Biomarker in Diabetic Retinopathy
Source: J Diabetes. 2025 Feb 19;17(2):e70058. doi: 10.1111/1753-0407.70058 (PMC11836613; doi:10.1111/1753-0407.70058)
Supplement: Supplementary file 1 — Data S1 Supporting Tables. [file JDB-17-e70058-s001.pdf]

Supplementary Table 1 Parameters of Study Sequences

Index	T_1-weighted	T_2-weighted	T_2-FLAIR	DWI
Philips Ingenia CX 3.0T MR with a 32-channel head coil				
TR (ms)	250	3100	100	2990
TE (ms)	2.3	103	11000	90
TI (ms)	NA	NA	2800	NA
FA (°)	75	90	110	90
Matrix size	432×432	512×512	384×384	192×192
Voxel size (mm^3)	0.53×0.53×5	0.45×0.45×5	0.60×0.60×5	1.20×1.20×5
B value (s/mm^3)	NA	NA	NA	1000
Slice thickness (ms)	5	5	5	5
Philips Achieva TX 3.0T MR with an 8-channel head coil				
TR (ms)	102	3712	6000	2384

TE (ms)	1.5	100	120	70
TI (ms)	NA	NA	2000	NA
FA (°)	80	90	90	90
Matrix size	400×400	384×384	384×384	400×400
Voxel size (mm^3)	0.57×0.57×6	0.60×0.60×6	0.60×0.60×6	0.57×0.57×6
B value (s/mm^3)	NA	NA	NA	1000
Slice thickness (ms)	6	6	6	6
Philips Multiva 1.5T MR with an 8-channel head coil				
TR (ms)	102	3537	8000	3000
TE (ms)	1.7	95	110	85
TI (ms)	NA	NA	2450	NA
FA (°)	80	90	90	90
Matrix size	640×640	512×512	336×336	192×192

Voxel size (mm^3)	0.36×0.36×6	0.45×0.45×6	0.68×0.68×6	1.20×1.20×6
B value (s/mm^3)	NA	NA	NA	1000
Slice thickness (ms)	6	6	6	6

TR =Repetition Time, TE =Echo Time, FA =Flip Angle, FOV =Field of View, TI =Inversion Time

Supplementary Table 2 Quantitative Analysis of CSVD Imaging Features of DR Group and NDR Group

CSVD parameters	total (n=365)	NDR (n=243)	DR (n=122)	Crude *P*	Model 1	Model 2	Model 3	Model 4	Model 5
**PVS**									
Total PVS	22 (14, 33)	22 (13, 32)	23 (16, 34)	0.164	0.156	0.16	0.132	0.238	0.197
Total PVS (L)	11 (7, 16)	11 (7, 16)	11 (7.25, 16)	0.542	0.498	0.516	0.461	0.614	0.622
Total PVS (R)	11 (7, 17)	10 (7, 16.5)	12.5 (8, 17.8)	0.070	0.175	0.171	0.131	0.251	0.147
Lobar PVS	12 (7, 19)	12 (7, 19)	12 (7, 18)	0.841	0.964	0.971	0.883	0.91	0.819
Lobar PVS (L)	6 (3, 9)	6 (3, 9)	5 (3, 8)	0.47	0.504	0.476	0.53	0.414	0.509
Lobar PVS (R)	6 (3, 10)	6 (3, 10)	6 (3, 10)	0.258	0.391	0.38	0.324	0.495	0.225
Frontal PVS (L)	3 (2, 6)	3 (2, 7)	3 (2, 5)	0.260	0.091	0.075	0.092	0.059	0.088
Frontal PVS (R)	3 (1, 6)	3 (1, 6)	3 (1, 6)	0.930	0.796	0.78	0.725	0.881	0.402
Parietal PVS (L)	1 (0, 1)	1 (0, 1)	1 (0, 2)	0.372	0.01*	0.009*	0.006*	0.011*	0.005*

Parietal PVS (R)	1 (0, 2)	1 (0, 2)	1 (0, 2)	0.360	0.801	0.806	0.761	0.962	0.942
Temporal PVS (L)	1 (0, 2)	1 (0, 2)	1 (0, 2)	0.477	0.245	0.242	0.331	0.354	0.429
Temporal PVS (R)	1 (1, 3)	1 (0.5, 2)	1 (1, 3)	0.116	0.028*	0.029*	0.017*	0.033*	0.025*
Occipital PVS (R)	12 (3.29)	7 (2.88)	5 (4.1)	0.538	NA	NA	NA	NA	NA
Deep PVS	10 (5, 14)	9 (5, 14)	10.5 (7, 16)	0.021*	0.022*	0.022*	0.02*	0.04*	0.05*
Deep PVS (L)	5 (2, 8)	5 (2, 7)	5 (3, 8.75)	0.054	0.032*	0.033*	0.031*	0.056	0.089
Deep PVS (R)	5 (2, 8)	4 (2, 7)	5 (3, 8)	0.039*	0.069	0.067	0.054	0.106	0.105
Basal ganglia PVS (L)	5 (2, 7)	5 (2, 7)	5 (3, 8)	0.047*	0.017*	0.017*	0.017*	0.033*	0.054
Basal ganglia PVS (R)	5 (2, 7)	4 (2, 7)	5 (3, 8)	0.043*	0.086	0.084	0.066	0.127	0.128
Thalamus PVS (L)	32 (8.77)	21 (8.64)	11 (9.02)	0.905	NA	NA	NA	NA	NA
Thalamus PVS (R)	31 (8.49)	16 (6.58)	15 (12.3)	0.065	NA	NA	NA	NA	NA
**DPVS**									
Total DPVS	4 (3, 7)	4 (2, 6)	5 (3, 7)	0.059	0.115	0.126	0.127	0.179	0.244

Total DPVS (L)	2 (1, 4)	2 (1, 3)	3 (2, 4)	0.010*	0.032*	0.036*	0.033*	0.04*	0.076
Total DPVS (R)	2 (1, 4)	2 (1, 3)	2 (1, 4)	0.576	0.407	0.425	0.455	0.61	0.603
Lobar DPVS	1 (0, 3)	1 (0, 3)	1 (0, 3)	0.516	0.292	0.316	0.317	0.402	0.304
Lobar DPVS (L)	1 (0, 2)	1 (0, 2)	1 (0, 1.75)	0.501	0.026*	0.029*	0.033*	0.053	0.025*
Lobar DPVS (R)	178 (48.77)	115 (47.33)	63 (51.64)	0.437	NA	NA	NA	NA	NA
Frontal DPVS (L)	127 (34.79)	79 (32.51)	48 (39.34)	0.196	NA	NA	NA	NA	NA
Frontal DPVS (R)	117 (32.05)	80 (32.92)	37 (30.33)	0.616	NA	NA	NA	NA	NA
Parietal DPVS (L)	58 (15.89)	38 (15.64)	20 (16.39)	0.852	NA	NA	NA	NA	NA
Parietal DPVS (R)	57 (15.62)	30 (12.35)	27 (22.13)	0.731	NA	NA	NA	NA	NA
Temporal DPVS (L)	41 (11.23)	25 (10.29)	16 (13.11)	0.420	NA	NA	NA	NA	NA
Temporal DPVS (R)	85 (23.29)	52 (21.4)	33 (27.05)	0.228	NA	NA	NA	NA	NA
Deep DPVS	3 (2, 5)	3 (2, 4)	3 (2, 5)	0.023*	0.019*	0.021*	0.02*	0.034*	0.093
Deep DPVS (L)	2 (1, 3)	1 (1, 2)	2 (1, 3)	**0.001***	**<0.001***	**<0.001***	**<0.001***	**<0.001***	**0.001***

Deep DPVS (R)	1 (1, 2)	1 (1, 2)	1 (0.25, 2)	0.728	0.623	0.648	0.625	0.79	0.974
Basal ganglia DPVS (L)	2 (1, 3)	1 (1, 2)	2 (1, 3)	**0.002***	**<0.001***	**<0.001***	**<0.001***	**<0.001***	**0.001***
Basal ganglia DPVS (R)	1 (1, 2)	1 (1, 2)	1 (0.25, 2)	0.759	0.661	0.689	0.664	0.823	0.941
**WMH**									
Volume (mm^3)	1158 (0, 10278)	680 (0, 8663)	2556 (65.5, 11267)	0.05*	0.31	0.384	0.343	0.355	0.364
Volumetric ratio	1.89 (0, 16.5)	1.10 (0, 14.0)	3.95 (0.1, 18.9)	0.051	--	0.028*	0.028*	0.101	0.068
**SSI**									
Total SSI	74 (20.27)	40 (16.46)	34 (27.87)	0.011*	NA	NA	NA	NA	NA
Total SSI (L)	30 (8.22)	21 (8.64)	9 (7.38)	0.678	NA	NA	NA	NA	NA
Total SSI (R)	50 (13.7)	22 (9.05)	28 (22.95)	**<0.001***	NA	NA	NA	NA	NA
Lobar SSI (R)	34 (9.32)	17 (7)	17 (13.93)	0.031*	NA	NA	NA	NA	NA
Frontal SSI (L)	12 (3.29)	7 (2.88)	5 (4.1)	0.538	NA	NA	NA	NA	NA
Frontal SSI (R)	19 (5.21)	9 (3.7)	10 (8.2)	0.068	NA	NA	NA	NA	NA

Temporal SSI (R)	10 (2.74)	5 (2.06)	5 (4.1)	0.274	NA	NA	NA	NA	NA
Deep SSI (R)	20 (5.48)	7 (2.88)	13 (10.66)	**0.002***	NA	NA	NA	NA	NA
Basal ganglia SSI (R)	17 (4.66)	5 (2.06)	12 (9.84)	**0.001***	NA	NA	NA	NA	NA
**Lacunes**									
Total Lacunes	30 (8.22)	17 (7)	13 (10.66)	0.23	NA	NA	NA	NA	NA

Some CSVD variables (right occipital PVS, thalamus PVS, right lobar DPVS, frontal DPVS, parietal DPVS, temporal DPVS, and all included parameters of SSI and lacunes) were analyzed as categorical variables while others were treated as continuous variables with a skewed distribution given the data distributions. Model 1 adjusted ICV. Model 2 is model 1 plus adjustment for sex, and age. Model 3 is model 2 plus adjustment for smoking habits and alcohol consumption. Model 4 is model 3 plus adjustment for hypertension and hyperlipidemia. Model 5 is model 4 plus adjustment for BMI and duration of diabetes.

* P <0.05; Bold P values indicate significance after FDR correction.

The volume (mm3) ratio didn't adjust ICV for it is derived data calculated by dividing the volume by ICV.

PVS =perivascular spaces, DPVS =dilated perivascular spaces, WMH =white matter hyperintensity, SSI =small subcortical infarcts.

Supplementary Table 3 Brain Volume between DR Group and NDR Group.

Brain volume, cm^3	total (n=365)	NDR (n=243)	DR (n=122)	Model 1 Padj	Model 2 Padj	Model 3 Padj	Model 4 Padj	Model 5 Padj
Frontal	147±14.9	147±14.5	147±15.7	0.010*	0.004*	0.008*	0.013*	0.045*
Frontal (L)	74.2±7.52	74.2±7.29	74.1±7.99	0.013*	0.005*	0.009*	0.016*	0.049*
Frontal (L)	72.7±7.5	72.7±7.38	72.7±7.78	0.011*	0.006*	0.012*	0.018*	0.050*
Medial Temporal	39.9±4.21	39.6±4.27	40.4±4.06	0.227	0.232	0.225	0.206	0.272
Medial Temporal (L)	19.7±2.11	19.5±2.09	19.9±2.12	0.242	0.271	0.278	0.285	0.358
Medial Temporal (R)	20.2±2.27	20.1±2.33	20.5±2.12	0.294	0.288	0.265	0.221	0.285
Lateral Temporal	65.9±7.49	65.5±7.28	66.6±7.86	0.642	0.705	0.723	0.632	0.306
Lateral Temporal (L)	32.9±3.88	32.7±3.84	33.4±3.94	0.465	0.468	0.517	0.435	0.2
Lateral Temporal (R)	32.9±3.8	32.8±3.63	33.3±4.11	0.908	0.965	0.995	0.929	0.552
Parietal	115±11.7	114±11	115±13	0.327	0.18	0.175	0.247	0.618

	Parietal (L)	56.7±5.89	56.6±5.57	57±6.5	0.267	0.139	0.161	0.224	0.57
	Parietal (R)	57.9±6	57.8±5.64	58.3±6.66	0.458	0.303	0.251	0.34	0.716
Occipital		46.1±5.17	46.3±5.34	45.9±4.84	0.051	0.051	0.04*	0.043*	0.048*
	Occipital (L)	22.4±2.58	22.5±2.66	22.4±2.42	0.158	0.165	0.162	0.171	0.138
	Occipital (R)	23.7±2.78	23.8±2.86	23.5±2.61	0.024*	0.023*	0.014*	0.016*	0.026*
Insula		13.3±1.48	13.4±1.46	13.3±1.52	0.099	0.111	0.09	0.155	0.548
	Insula (L)	6.72±0.73	6.72±0.73	6.73±0.75	0.245	0.27	0.255	0.42	0.965
	Insula (R)	6.63±0.8	6.64±0.78	6.62±0.85	0.092	0.101	0.077	0.109	0.427
Cingulum		17.6±2.21	17.5±2.13	17.9±2.37	0.56	0.62	0.722	0.612	0.338
	Cingulum (L)	8.84±1.24	8.8±1.23	8.92±1.28	0.966	0.833	0.784	0.851	0.772
	Cingulum (R)	8.79±1.23	8.72±1.18	8.94±1.32	0.343	0.351	0.437	0.35	0.249
Basal ganglia		19.9±2.86	19.8±2.79	19.9±2.99	0.746	0.657	0.681	0.702	0.898
	Basal ganglia (L)	9.69±1.47	9.67±1.46	9.73±1.52	0.817	0.687	0.697	0.717	0.864

Basal ganglia (R)	10.2±1.51	10.2±1.48	10.2±1.57	0.76	0.72	0.761	0.78	0.977
Subcortical GM	34.7±4.16	34.7±4.08	34.6±4.33	0.157	0.062	0.071	0.098	0.167
Subcortical GM (L)	17.4±2.22	17.5±2.16	17.4±2.33	0.155	0.055	0.067	0.092	0.126
Subcortical GM (R)	17.2±2.09	17.2±2.06	17.2±2.15	0.206	0.115	0.123	0.158	0.29
Corpus callosum	3.49±0.45	3.47±0.46	3.52±0.42	0.794	0.944	0.973	0.921	0.985
Cerebral WM	421±49.9	419±49.6	426±50.4	0.834	0.994	0.929	0.78	0.657
Cerebral WM (L)	210±24.7	209±24.6	212±24.9	0.868	0.935	0.968	0.828	0.66
Cerebral WM (R)	211±25.4	210±25.2	213±25.7	0.828	0.979	0.921	0.764	0.69

Data was obtained from the Independent-samples T-test and multivariate analysis of variance. Model 1 adjusted ICV. Model 2 is model 1 plus adjustment for sex, and age. Model 3 is model 2 plus adjustment for smoking habits and alcohol consumption. Model 4 is model 3 plus adjustment for hypertension and hyperlipidemia. Model 5 is model 4 plus adjustment for BMI and duration of diabetes.

Due to space limitations, data showing significant differences were presented. To ensure consistency between Tables 4 and 5, the analysis data for subcortical GM volume and light subcortical GM volume were also included. Additional details can be found in the supplementary materials.

* $P < 0.05$.

GM = gray matter, WM =white matter

Supplementary Table 4 Brain Volumetric Ratios between DR Group and NDR Group

Brain volumetric ratio, %	total (n=365)	NDR (n=243)	DR (n=122)	Model 1 P	Model 2 P_{adj}	Model 3 P_{adj}	Model 4 P_{adj}	Model 5 P_{adj}
Frontal	10.5±0.52	10.5±0.52	10.4±0.52	0.014*	0.003*	0.006*	0.01*	0.036*
Frontal (L)	5.3±0.27	5.32±0.27	5.25±0.28	0.017*	0.003*	0.006*	0.012*	0.037*
Frontal (R)	5.19±0.27	5.22±0.27	5.15±0.26	0.02*	0.005*	0.01*	0.015*	0.050*
Medial Temporal	2.85±0.2	2.84±0.21	2.87±0.18	0.235	0.326	0.32	0.3	0.365
Medial Temporal (L)	1.41±0.11	1.4±0.11	1.41±0.1	0.312	0.411	0.418	0.432	0.496
Medial Temporal (R)	1.44±0.11	1.44±0.12	1.45±0.1	0.243	0.335	0.317	0.272	0.333
Lateral Temporal	4.7±0.3	4.7±0.32	4.72±0.28	0.543	0.707	0.711	0.623	0.3
Lateral Temporal (L)	2.35±0.17	2.35±0.18	2.36±0.15	0.372	0.491	0.529	0.443	0.2
Lateral Temporal (R)	2.35±0.16	2.35±0.16	2.35±0.15	0.827	0.991	0.967	0.898	0.538
Parietal	8.19±0.41	8.2±0.4	8.16±0.44	0.33	0.163	0.154	0.214	0.583

Parietal (L)	4.05±0.22	4.06±0.21	4.03±0.24	0.275	0.14	0.156	0.209	0.562
Parietal (R)	4.14±0.21	4.14±0.21	4.12±0.22	0.454	0.254	0.207	0.282	0.654
Occipital	3.3±0.28	3.32±0.28	3.26±0.29	0.061	0.046*	0.035*	0.038*	0.047*
Occipital (L)	1.6±0.15	1.61±0.15	1.59±0.15	0.173	0.143	0.135	0.147	0.129
Occipital (R)	1.7±0.15	1.71±0.15	1.67±0.16	0.031*	0.023*	0.013*	0.015*	0.026*
Insula	0.95±0.07	0.96±0.07	0.95±0.06	0.094	0.077	0.063	0.114	0.461
Insula (L)	0.48±0.04	0.48±0.04	0.48±0.03	0.223	0.201	0.191	0.326	0.852
Insula (R)	0.47±0.04	0.48±0.04	0.47±0.04	0.094	0.072	0.055	0.083	0.357
Cingulum	1.26±0.1	1.26±0.1	1.26±0.11	0.457	0.61	0.704	0.598	0.302
Cingulum (L)	0.63±0.07	0.63±0.06	0.63±0.07	0.858	0.925	0.882	0.943	0.653
Cingulum (R)	0.63±0.06	0.63±0.06	0.63±0.06	0.314	0.38	0.467	0.374	0.25
Basal ganglia	1.42±0.18	1.42±0.19	1.41±0.18	0.572	0.426	0.438	0.457	0.658
Basal ganglia (L)	0.69±0.1	0.7±0.1	0.69±0.1	0.669	0.499	0.5	0.519	0.677

Basal ganglia (R)	0.73±0.1	0.73±0.1	0.72±0.1	0.577	0.462	0.489	0.506	0.76
Subcortical GM	2.48±0.25	2.49±0.25	2.45±0.24	0.107	0.026*	0.03*	0.043*	0.086
Subcortical GM (L)	1.25±0.14	1.26±0.13	1.23±0.14	0.113	0.026*	0.031*	0.044*	0.069
Subcortical GM (R)	1.23±0.13	1.24±0.13	1.22±0.12	0.138	0.052	0.055	0.073	0.159
Corpus callosum	0.25±0.03	0.25±0.03	0.25±0.03	0.872	0.945	0.92	0.98	0.917
Cerebral_WM	30±1.85	30±1.89	30.1±1.78	0.496	0.76	0.699	0.58	0.492
Cerebral_WM (L)	15±0.93	15±0.94	15±0.9	0.529	0.815	0.736	0.625	0.488
Cerebral_WM (R)	15.1±0.95	15±0.98	15.1±0.9	0.489	0.738	0.693	0.566	0.529

Data was obtained from the Independent-samples T-test and multivariate analysis of variance. Model 1 is the unadjusted model. Model 2 is model 1 plus adjustment for sex and age. Model 3 is model 2 plus adjustment for smoking habits and alcohol consumption. Model 4 is model 3 plus adjustment for hypertension and hyperlipidemia. Model 5 is model 4 plus adjustment for BMI and duration of diabetes.

* P <0.05.
